# DNA methylation regulates the expression of the negative transcriptional regulators *ID2* and *ID4* during OPC differentiation

**DOI:** 10.1007/s00018-021-03927-2

**Published:** 2021-09-05

**Authors:** Assia Tiane, Melissa Schepers, Renzo Riemens, Ben Rombaut, Patrick Vandormael, Veerle Somers, Jos Prickaerts, Niels Hellings, Daniel van den Hove, Tim Vanmierlo

**Affiliations:** 1 Department of Neuroscience, Biomedical Research Institute, Faculty of Medicine and Life Sciences, Hasselt University, Hasselt, Belgium; 2 Department Psychiatry and Neuropsychology, School for Mental Health and Neuroscience, Maastricht University, Maastricht, Netherlands; 3 University MS Center (UMSC) Hasselt-Pelt, Hasselt, Belgium; 4 Institute of Human Genetics, Julius Maximilians University, Wuerzburg, Germany; 5 Department of Immunology and Infection, Biomedical Research Institute, Faculty of Medicine and Life Sciences, Hasselt University, Hasselt, Belgium; 6 Department of Psychiatry, Psychosomatics and Psychotherapy, University of Wuerzburg, Wuerzburg, Germany

**Keywords:** Oligodendrocyte, Oligodendrocyte precursor cell, Methylation, Myelination, ID2, ID4

## Abstract

The differentiation of oligodendrocyte precursor cells (OPCs) into myelinating oligodendrocytes is the prerequisite for remyelination in demyelinated disorders such as multiple sclerosis (MS). Epigenetic mechanisms, such as DNA methylation, have been suggested to control the intricate network of transcription factors involved in OPC differentiation. Yet, the exact mechanism remains undisclosed. Here, we are the first to identify the DNA-binding protein inhibitors, *Id2* and *Id4,* as targets of DNA methylation during OPC differentiation. Using state-of-the-art epigenetic editing via CRISPR/dCas9-DNMT3a, we confirm that targeted methylation of *Id2/Id4* drives OPC differentiation. Moreover, we show that in the pathological context of MS, methylation and gene expression levels of both *ID2* and *ID4* are altered compared to control human brain samples. We conclude that DNA methylation is crucial to suppress *ID2* and *ID4* during OPC differentiation, a process that appears to be dysregulated during MS. Our data do not only reveal new insights into oligodendrocyte biology, but could also lead to a better understanding of CNS myelin disorders.

**Supplementary Information:**

The online version contains supplementary material available at 10.1007/s00018-021-03927-2.

## Introduction

Oligodendrocytes are derived from a pool of proliferating oligodendrocyte precursor cells (OPCs) that exit the cell cycle and differentiate into mature oligodendrocytes, the myelinating cells of the central nervous system (CNS) [1, 2]. This differentiation process is a prerequisite for myelin formation and is strictly coordinated by a complex interplay between extracellular signals, intracellular transcription factors, and epigenetic mechanisms [3, 4]. Myelin genes are defined as genes that code for essential proteins of the myelin sheath, such as myelin basic protein (*MBP*), proteolipid protein (*PLP*), myelin oligodendrocyte glycoprotein (*MOG*) and myelin-associated glycoprotein (*MAG*). The expression of these myelin genes during OPC differentiation is regulated by an upstream transcriptional network. This myelin regulatory network is composed of positive regulators, which promote myelin gene expression, and negative regulators, which repress the expression of myelin genes and OPC differentiation, as reviewed more in detail by Tiane et al*.* [5, 6]. As such, tight control of both positive and negative upstream regulators is required to orchestrate OPC differentiation during myelin formation.

In many neurodegenerative diseases, such as multiple sclerosis (MS), damaging insults result in demyelination of certain axons, leaving the affected neurons dysfunctional and vulnerable to atrophy [7]. Endogenous remyelination is therefore crucial to restore the myelin sheath and prevent further neurodegeneration [8]. However, for reasons still not entirely elucidated, these remyelination mechanisms become insufficient as the disease progresses toward the chronic stage of MS, or with age in general [9, 10]. Evidence suggests that a significant part of this remyelination failure can be attributed to an impaired OPC differentiation capacity [10]. As such, OPCs are present within chronically demyelinated non-fibrotic MS lesions of MS patients, yet they seem to be unable to differentiate into myelinating oligodendrocytes [11, 12]. This differentiation and remyelination block is not observed in the early stages of MS, which suggests that changes in the micro-environment, such as accumulated lesion damage due to chronic inflammation, could influence OPC functioning [13–15]. Thus, to further comprehend the exact mechanisms causing remyelination failure, we should first gain a better understanding of the biology behind OPC differentiation.

Over recent years, focus has shifted towards the specific involvement of epigenetic mechanisms underlying OPC differentiation and (re)myelination. For example, a large body of evidence has shown that histone modifications are essential to regulate the transcriptional control of myelin genes during OPC differentiation [6, 16–18]. Emerging data also suggest that other epigenetic mechanisms, such as DNA methylation, strongly influence OPC cell fate commitment and (re)myelination. For instance, research has proven the DNA methyltransferase 1 (DNMT1) enzyme to be essential during developmental myelination, while DNMT3a plays a dominant role in adult remyelination after injury [19, 20]. Moreover, ten–eleven translocation (TET) enzymes, responsible for DNA hydroxymethylation and DNA demethylation, have shown to be differentially regulated during oligodendrocyte development and remyelination [21, 22]. These insights have shed new light on the process of oligodendrocyte development and might unravel new promising strategies to boost OPC differentiation. Nevertheless, which genes are actually targeted by the DNA methylation enzymes during OPC differentiation remains undisclosed.

Accordingly, we hypothesized that the upstream transcriptional regulators of myelin gene expression are themselves regulated by DNA methylation during OPC differentiation. In this study, we show that inhibition of DNA methylation in primary OPCs, by means of incorporation of 5-azacytidine (5-AZA), leads to a decreased OPC differentiation rate, accompanied by an increased expression of *Id2* and *Id4,* two negative transcriptional regulators of myelin genes. Furthermore, we observed that both the *Id2* and *Id4* promotors are hypermethylated during OPC differentiation, which is, in turn, negatively correlated with their gene expression levels. Moreover, CRISPR-dCas9-DNMT3a based targeted methylation of the promoter region of either *Id2* or *Id4* successfully inhibited their expression and boosted OPC differentiation and myelin gene expression. Interestingly, the promoter region of both genes was shown to be hypomethylated in chronically demyelinated inactive lesions of MS patients. To our knowledge, this is the first study that establishes the intricate relationship between DNA methylation of *Id2* and *Id4* and OPC differentiation.

## Materials and methods

### Primary OPC cultures

All in vitro mouse experiments were approved by the Hasselt University Ethics Committee for Animal Experiments. Primary mouse OPCs were obtained from mixed glial cultures, using the standard shake-off method (70). In brief, cortices were isolated from postnatal day 0 mice and cells were enzymatically dissociated by incubation with papain (3 U/ml, diluted in Dulbecco’s Modified Eagle Medium (DMEM) containing 1 mM l-cystein; Sigma-Aldrich, Bornem, Belgium) for 20 min. Mixed glial cells were maintained in DMEM (Sigma-Aldrich), supplemented with 50U/ml penicillin and 50 mg/ml streptomycin (P/S; Invitrogen, Merelbeke, Belgium) and 10% heat-inactivated fetal calf serum (FCS; Hyclone, Erebodegem, Belgium) on poly-l-lysine-coated (5 µg/ml, Sigma-Aldrich) culture flasks. Cells were kept at 37 °C in a humidified atmosphere of 8.5% CO2. From the seventh day, cells were maintained in culture medium, supplemented with bovine insulin (5 µg/ml; Sigma-Aldrich) to stimulate OPC formation within the mixed glial cultures. On day 14, the cells were shaken using an orbital shaker at 75 rpm and 37 °C for 45 min to detach the microglial layer. A second shake-off was performed for 16 h at 250 rpm, after which the OPC-enriched supernatant was collected, incubated for 20 min on a Petri dish and centrifuged on 300×*g* for five minutes. All cell cultures had a purity above 95%. OPCs were seeded onto 24-well plates and maintained in DMEM medium (+ 10% FCS and 1% P/S) or differentiation medium (DMEM medium, supplemented with 0.5% P/S, 2% horse serum, 0.3 mM transferrin, 0.1 mM putrescin, 0.02 mM progesterone, 0.2 µM sodium selenite, 0.5 µM triiodothyronin, 0.8 mM bovine insulin, 0.5 mM L-thyroxine, 2% B27 supplement; all from Sigma-Aldrich except for P/S, Invitrogen, and B27, in house production as described by Chen et al. [23]), depending on the experiment.

#### 5-AZA treatment

Primary OPC cultures were kept in a proliferating state by addition of 5 ng/µl platelet-derived growth factor α (PDGFα; Peprotech, Rocky Hill, USA) to the DMEM culture medium, and were treated for three consecutive days with 1 µM 5-AZA (Sigma-Aldrich) or DMSO (Sigma-Aldrich) as a vehicle control. After 3 or 6 days of rest in differentiation medium, OPCs were either lysated for RNA isolation or fixed on coverslips to assess their morphology and protein expression via immunofluorescence.

#### Transfection

The pdCas9-DNMT3A-PuroR plasmid was a gift from Vlatka Zoldoš (Addgene plasmid #71,667). The catalytically inactive pdCas9-DNMT3A-PuroR vector (Addgene plasmid #71,684) was taken along as a negative control. Plasmids were transfected into primary OPCs 24 h after seeding, using the OZ Biosciences NeuroMag Transfection Reagent (Bio-connect, Huissen, The Netherlands), following the manufacturer’s instructions. In brief, 500 ng of plasmid DNA was diluted in 50 µl DMEM medium, added to 1.75 µl NeuroMag reagent and incubated for 20 min on room temperature. DNA/NeuroMag complexes were dropwise added to primary OPC cultures (200,000 cells/well), maintained in P/S free differentiation medium, and placed on a magnetic plate for 30 min in an 8.5% CO_2_ incubator. Two days after transfection, transfected cells were selected for 72 h with 5 µg/ml puromycin (Invivogen, Toulouse, France), a dose-optimized concentration of puromycin with 100% mortality in non-transfected cells. OPCs were then kept in standard differentiation medium until further experiments.

### CRISPR-dCas9-DNMT3a plasmids

#### Design guide RNA

The promoter regions of *Id2* and *Id4* were exported from the Ensembl database and were scanned for CpG islands using the default CpG islands track in the UCSC Genome Browser. Specific guide RNAs (sgRNAs) were designed to induce methylation within the promoter region of the *Id2* (chr12:25.097.141-25.097.740) and *Id4* (chr13:48.260.628-48.261.228) genes using Benchling software^®^. For each gene, the guide with the lowest off-target prediction was used (Supplementary Table S1). Guides were synthesized as oligo’s with overhangs to fit into the BbsI restriction gap and an additional guanine for increased transcriptional efficiency.

#### sgRNA cloning and transformation

Plasmid DNA (1 µg; Addgene plasmids #71,667 and #71,684) was incubated overnight on 37 °C with 40U BbsI restriction enzyme (Bioké, Leiden, The Netherlands). Enzyme inactivation was performed by incubation on 65 °C for 20 min, after which the samples were immediately loaded on an agarose gel (1%). The open vector was extracted from the gel, using the PCR and gel clean-up kit (Macherey–nagel, Düren, Germany), according to the manufacturer’s instructions. Annealed sgRNAs were ligated with the T4 DNA Ligase buffer and enzyme system (Bioké) into the linearized vector in a 5:1 insert to vector molar ratio. The ligated product was then transformed into NEB^®^ 5-alpha Competent E. coli cells (Bioké) and plated out on LB-agar plates, supplemented with ampicillin (Amp; 100 mg/ml). Suitable colonies were propagated overnight in LB-Amp medium. Plasmids were extracted using the NucleoBond^®^ Xtra Midi kit, according to the manufacturer’s protocol (Macherey–Nagel). SANGER sequencing was carried out on purified plasmid vector to validate the sgRNA incorporation.

### Immunostaining

#### Immunocytochemistry

Primary OPCs were fixed in 4% paraformaldehyde (PFA) for 30 min at room temperature at day 6 post 5-AZA treatment or day 9 post transfection. Aspecific binding was blocked for 30 min with 1% bovine serum albumin (BSA) in 0.1% PBS-T, followed by incubation with primary antibodies (Supplementary Table S2) for four hours at room temperature. After three washing steps with PBS, cells were incubated with Alexa 488- or Alexa 555-conjugated secondary antibody (Supplementary Table S2) for one hour. Nuclei were counterstained with 4′6-diamidino-2-phenylindole (DAPI; Sigma-Aldrich). Coverslips were mounted with Dako mounting medium (Dako, Carpinteria, USA) and analyzed using a fluorescence microscope (Leica DM2000 LED). Images were quantified using Fiji, ImageJ software [3 pictures per coverslip]. The percentage positive for MBP or O4 was quantified and divided by the percentage positive for DAPI, to correct for cell numbers. Process length was determined by measuring the longest process per cell in pixels.

#### Immunohistochemistry

Human post-mortem brain tissue was obtained through the Netherlands Brain Bank (www.brainbank.nl) (demographic characteristics described in Table 1). MS lesion sections were characterized for demyelination, inflammation, and presence of OPCs by immunohistochemistry. Sections were fixed in ice-cold aceton for 10 min and blocked for 30 min with the Dako Protein Block (Dako) at room temperature. Primary antibodies (Supplementary Table S2) were added for overnight incubation at 4 °C. After repeated washing steps, sections were incubated with horseradish peroxidase (HRP)-conjugated EnVision + Dual Link System (Dako) for 30 min. Unbound antibodies were washed away with PBS and sections were incubated with the DAKO 3,3'-diaminobenzidine (DAB) solution (Dako) for color development. Nuclei were counterstained with haematoxylin for 2 min. Following extensive washing in tap water, sections were dehydrated in increasing concentrations of ethanol (70%, 80%, 95%, and 100%) and xylene. Oil Red O (ORO) staining was used to stain for lipid containing phagocytes within MS lesions. Brain sections were stained in 0.3% ORO (Sigma-Aldrich) for 10 min, and counterstained with haematoxylin for 1 min. The stained tissues were mounted with DPX Mountant (Leica Microsystems, Wetzelar, Germany) and visualized with a Leica DM2000 LED Microscope equipped with a Leica MC170 HD Camera (Leica Microsystems).

**Table 1:** Demographic characteristics of the cohort

Characteristics	Non-neurologic controls	MS patients
Gender (male/female)	4/6	4/6
Age, mean (SD)	65.7 (8.90)	64.7 (9.64)
Disease diagnosis (PPMS/PRMS/SPMS/unspecified)	n.a	3/1/4/2
PMI, mean (SD)	9.12 (1.87)	9.72 (4.09)

*MS* multiple sclerosis, *PPMS* Primary Progressive MS, *PRMS* Primary Relapsing MS, *SPMS* Secondary Progressive MS, *PMI* post-mortem interval, *SD* standard deviation, *n.a*. not applicable

### Quantitative PCR

Total RNA was isolated from cells or brain tissue, using the RNeasy mini kit (Qiagen, Venlo, the Netherlands), according to the manufacturer’s instructions. RNA concentration and quality were analyzed with a Nanodrop spectrophotometer (Isogen Life Science, Leiden, The Netherlands). RNA was reverse-transcribed using the qScript cDNA Supermix kit (Quanta, Leuven, Belgium). qPCR was performed to analyze gene expression, using the Applied Biosystems QuantStudio 3 Real-Time PCR System (Life Technologies, Gent, Belgium). The reaction mixture consisted of SYBR Green master mix (Life Technologies), 10 µM forward and reverse primers (Integrated DNA Technologies, Leuven, Belgium), nuclease-free water and cDNA template (12.5 ng), up to a total reaction volume of 10 µl. The primer pairs used for amplification are listed in Supplementary Table 3. Results were analyzed by the comparative Ct method and were normalized to the most stable housekeeping genes (*Rpl13a/ Yhwaz* for murine OPCs and *YWHAZ/TBP* for human brain samples), determined by GeNorm.

### Genomic DNA isolation and pyrosequencing

Genomic DNA was extracted from transfected OPCs and bisulfite-converted, using the Zymo Research EZ DNA Methylation-Direct Kit (BaseClear Lab Products, Leiden, The Netherlands). For human brain samples, genomic DNA was extracted using a standard chloroform–phenol extraction and ethanol-precipitation method. Human genomic DNA purity was assessed by measuring the A260/A280 ratio using a NanoDrop (Isogen Life Science). A total of 500 ng human genomic DNA was subsequently bisulfite-converted using the EZ DNA Methylation-Direct Kit (Zymo Research). PCR primers were designed using the PyroMark^®^ Assay Design 2.0 software (Qiagen, Supplementary Table 4). Product amplification was performed using the following reaction mixture: 1X Buffer with 20 mM MgCl2 (Roche, Bornem, Belgium), 10 mM dNTP mix (Roche), 5 µM forward and reverse primers (Metabion AG, Planegg/Steinkirchen, Germany), 1U FastStart Taq DNA Polymerase (Roche), bisulfite-converted DNA and nuclease-free water to a total volume of 25 µl. PCR cycling was performed as follows: initial denaturation for 5 min at 95 °C, 50 cycles of 30 s at 95 °C, 30 s at 56 °C (mouse *Id2*), 60 °C (mouse *Id4*) or 58 °C (human *ID2* and *ID4*) and 1 min at 72 °C; final extension for 7 min at 72 °C. PCR amplicons were sequenced using the Pyromark™ Q48 instrument (Qiagen) with the PyroMark Q48 Advanced CpG Reagents (Qiagen), according to the manufacturer’s protocol and quantified with the Pyromark™ Q48 Autoprep software. The human assays for *ID2* and *ID4* were tested for their sensitivity using the EpiTect PCR Control DNA Set (Qiagen). Mouse DNA was manually demethylated by two subsequent whole genome amplification steps using the illustra GenomiPhi™ V2 DNA Amplification Kit (GE Healthcare BioScience, Uppsala, Sweden). After the first (10 µl volume) and the second elution (20 µl volume), the DNA was purified with the DNA Clean & ConcentratorTM-5 Kit (Zymo Research) according to the manufacturer’s instructions. A fully methylated “100% Universal Methylated Mouse DNA Standard” (Zymo Research) was commercially acquired. The mouse assays for Id2 and Id4 were tested for their sensitivity using the aforementioned standards.

### Statistical analysis

Statistical analysis was performed using GraphPad Prism 9.0.0 software (GraphPad software Inc., CA, USA). Differences between group means were determined using an unpaired t test for normally distributed data and a Mann–Whitney test for not normally distributed data. Differences within samples were assessed using one-sample *t* test for normally distributed data or Wilcoxon test for not normally distributed data. Correlation analysis was performed by the Spearman’s rank correlation test. Differences in methylation at different CpG sites were determined using a two-way repeated measures ANOVA with Šídák's multiple comparisons test. All data are depicted as mean ± SEM, *p ≤ 0.05, **p < 0.01, ***p < 0.001, ****p < 0.0001.

## Results

### Inhibition of DNA methylation prevents OPC differentiation and is associated with an increased expression of the negative regulators *Id2* and *Id4*

**Fig. 1:**
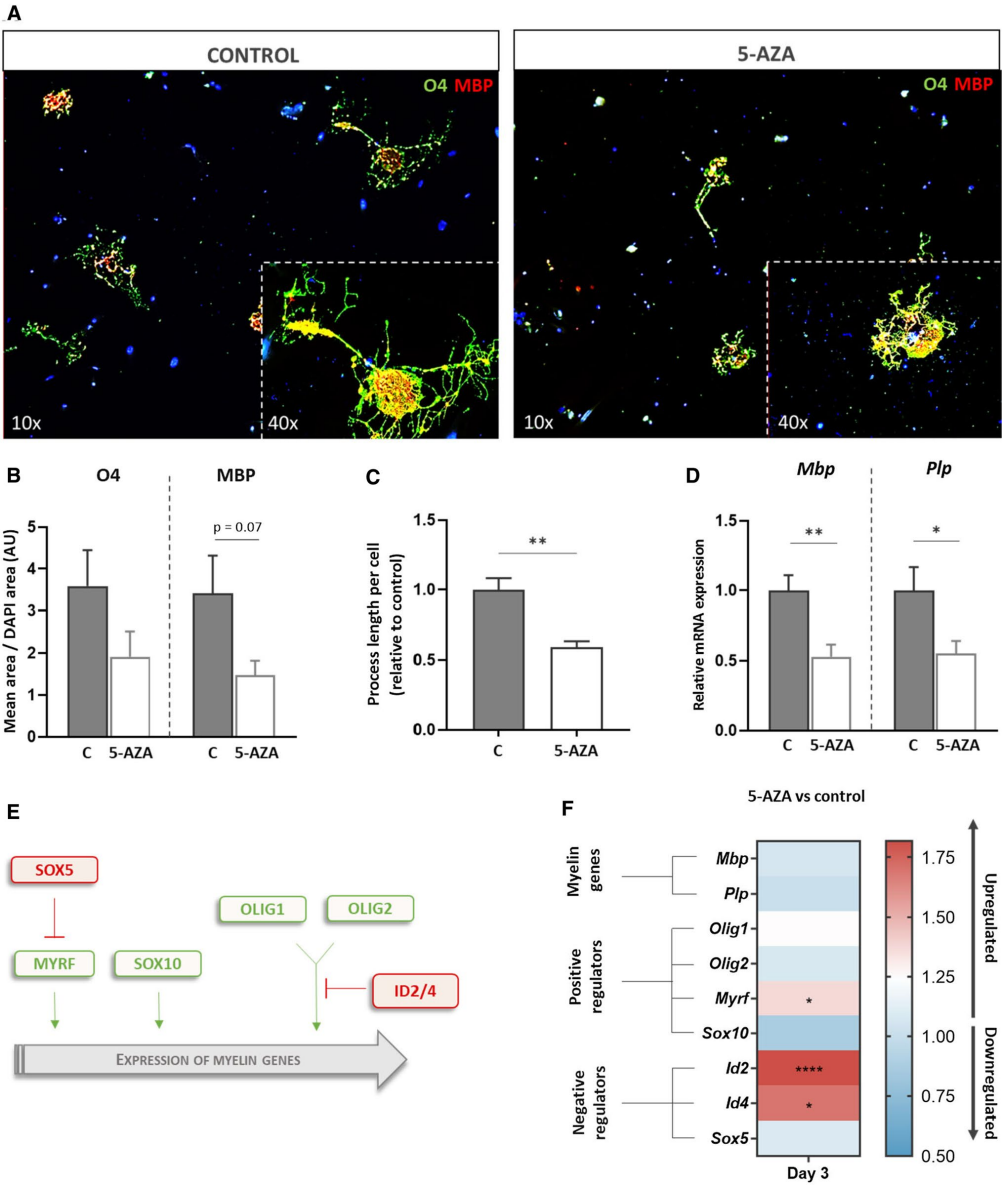
5-AZA inhibits OPC differentiation and is associated with an increased expression of *Id2* and *Id4*. **A**–**C** Representative images and quantification (fluorescence area and process length) of primary OPC cultures treated with 1 µM 5-AZA or DMSO as a vehicle control. **D-F** mRNA expression analysis of myelin genes and the upstream regulatory transcriptional network of 5-AZA-treated primary OPCs, compared to control-treated cells 6 days (**D**) or 3 days (**F**) post treatment. Data are corrected for the most stable housekeeping genes (*Rpl13a* and *Cypa*) and are represented as mean + SEM, *n* = 5, unpaired *t* test, *p < 0.05, **p < 0.01, and ****p < 0.0001. 5-AZA 5-azacytidin, O4 oligodendrocyte surface marker claudin-11, MBP/*Mbp* myelin basic protein, *Plp* myelin proteolipid protein, *Olig1/2* oligodendrocyte transcription factor 1/2, *Sox10/5* SRY-related HMG-box protein 10/5, *Id2/4* inhibitor of DNA-binding protein 2/4

We pharmacologically treated primary mouse-derived OPCs with 1 µM 5-AZA (Supplementary Fig. 1), a cytidine analog that prevents DNA methylation transfer during cell divisions, to assess its effect on subsequent cellular differentiation. Immunocytochemical analysis with O4 antibody (a marker for pre-mature oligodendrocytes) and a myelin basic protein (MBP) antibody (a marker for differentiated oligodendrocytes) demonstrated a decreased rate of OPC differentiation 6 days after 5-AZA treatment, compared to vehicle-treated OPCs (Fig. 1A–B). Morphological assessment of the cells showed that 5-AZA-treated OPCs mainly retained a simple morphology, while vehicle-treated OPCs differentiated into oligodendrocytes with longer process extensions (Fig. 1C). In line with this, gene expression analysis confirmed a reduced expression of myelin genes at the same time point (Fig. 1D). Subsequently, we aimed to address whether the myelin transcriptional regulatory network is already affected at an early stage of inhibition of DNA methylation. To this end, the gene expression of myelin regulatory pathway was measured 3 days after 5-AZA or vehicle control treatment (Fig. 1E, F). Interestingly, while the expression of most positive regulators was unaltered at this stage, the negative regulators *Id2* and *Id4* showed an increased expression upon treatment with the DNA methylation inhibitor (Fig. 1F).

### *Id2*/*Id4* promoter methylation is negatively correlated with its gene expression levels during OPC differentiation

**Fig. 2:**
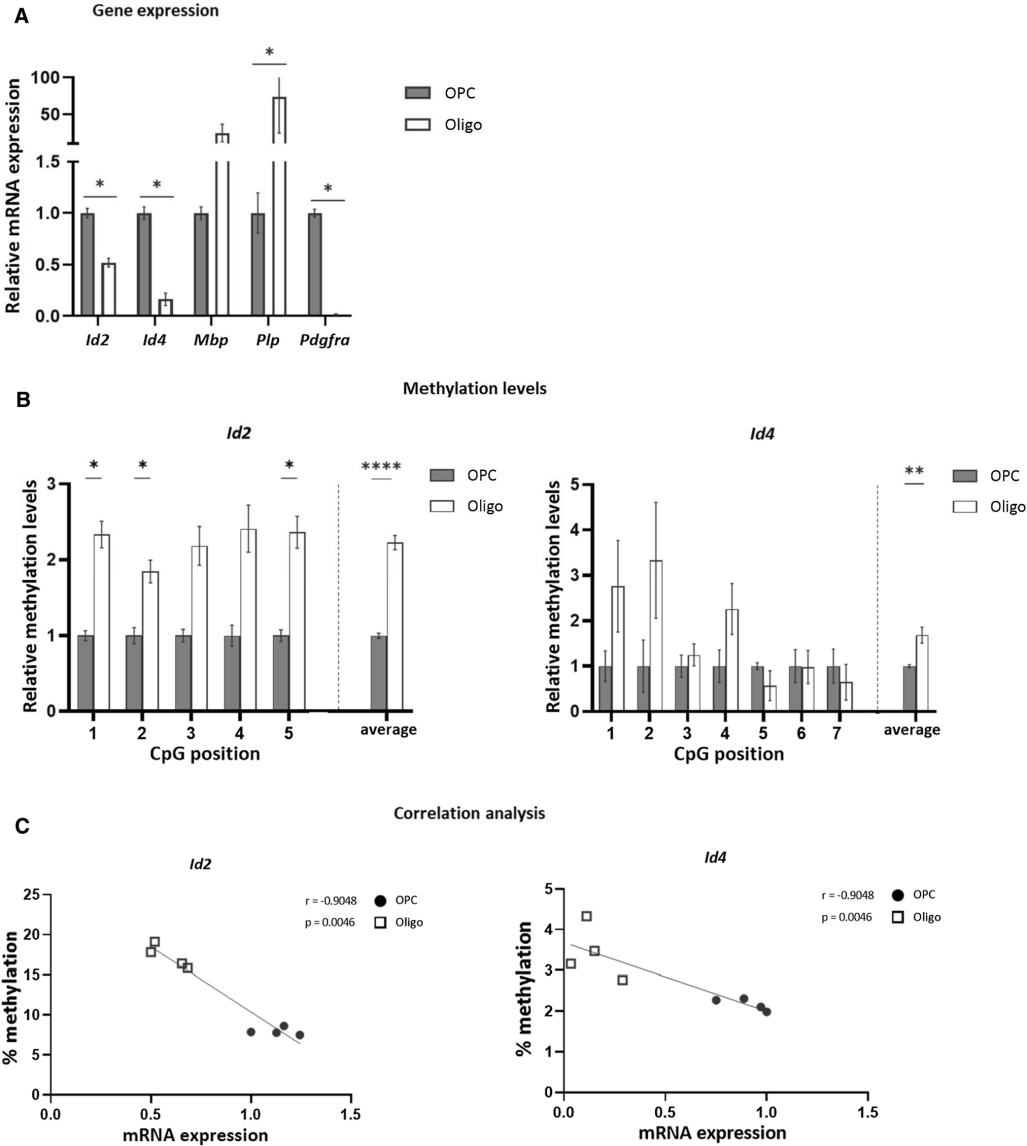
Decrease in *Id2/Id4* expression from OPC to oligodendrocyte stages is associated with an increased methylation profile. **A** Gene expression analysis in primary murine OPCs (day 0) and differentiated oligodendrocytes (day 12) of *Id2/4* and OPC (*Pdgfra*) and oligo (*Mbp, Plp*) markers. Data are corrected for the most stable housekeeping genes (*Pgk-1* and *Cypa*), *n* = 4, Mann–Whitney test. **B** Methylation analysis of the promoter region of both *Id2* and *Id4*, measured in cells from the same batches (*n* = 4, two-way repeated measures ANOVA with Šídák's multiple comparisons test). **C** Spearman’s correlation analysis between expression (**A**) and methylation (**B**) levels (*n* = 8). Data are represented as mean + SEM, **p < 0.01, ****p < 0.0001. *Oligo* oligodendrocytes

As we observed an increased expression of the helix–loop–helix (HLH) inhibitory transcription factors *Id2* and *Id4* upon DNA methylation inhibition, we next investigated their expression and methylation profile at the different stages of in vitro OPC differentiation. The expression of both *Id2* and *Id4* decreased significantly during the differentiation of murine OPCs into mature oligodendrocytes (Fig. 2A). Interestingly, average methylation within the promoter region of *Id2*/*Id4* was increased in mature oligodendrocytes compared to OPCs (Fig. 2B). Furthermore, the expression and methylation levels showed a strong negative correlation, suggesting that DNA methylation is necessary for the transcriptional regulation of both genes during OPC differentiation (Fig. 2C).

### Targeted hypermethylation of *Id2*/*Id4* using CRISPR-dCas9-DNTM3A decreases their expression and stimulates OPC differentiation

**Fig. 3:**
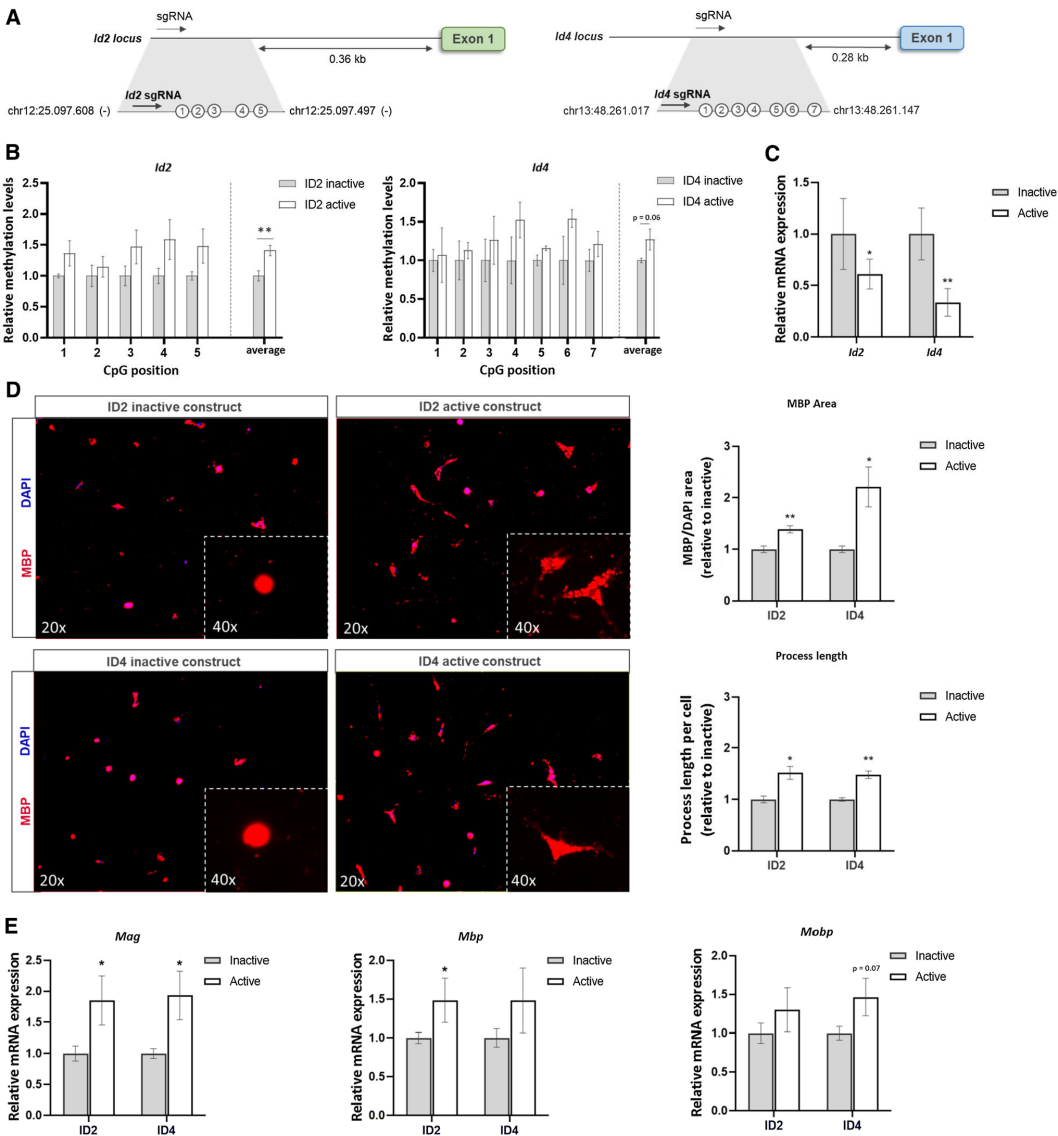
Targeted methylation of *Id2*/*Id4* with CRISPR-dCas9 results in lower gene expression and boosts OPC differentiation. **A** Primary OPCs were transfected with a CRISPR-pdCas9-DNMT3a vector targeting the *Id2* or *Id4* promoter region. Numbers reflect the number of CpG sites measured for methylation. **B** Methylation analysis confirms targeted methylation of the promotor of both genes 6 days after transfection, compared to control-transfected cells (*n* = 5, two-way repeated measures ANOVA with Šídák's multiple comparisons test). **C** Gene expression analysis showed lower expression of both genes. Data are corrected for the most stable housekeeping genes (*Rpl13a* and *Ywhaz*), *n* = 5, one-sample *t* test. **D** Representative images (20 × MBP and DAPI, 40 × MBP) and quantification (fluorescence area and average process length) of OPCs, 9 days post transfection (*n* = 5, one-sample *t* test). **E** Myelin gene (*Mag, Mbp, Mobp*) expression was increased after targeted methylation of either *Id2* or *Id4* (*n* = 7–8, Wilcoxon test). Data are represented as mean ± SEM, *p < 0.05, **p < 0.01. *sgRNA* guide RNA, *inactive* catalytic inactive pdCas9-DNMT3A-PuroR vector (control), *active* catalytic active pdCas9-DNMT3A-PuroR vector

To validate the causal relationship between *Id2*/*Id4* methylation and OPC differentiation, we made use of recently developed CRISPR-pdCas9-DNMT3a epigenetic editing plasmids [24]. Guide RNAs were designed to target specific CpG-rich regions within the promoter of *Id2* or *Id4* (Fig. 3A). In this way, the inactivated Cas9 protein (dCas9), which was attached to the catalytic domain of DNMT3a, was guided to the promoter region of our target genes, inducing methylation at the associated CpG sites. At day 6 post transfection, primary OPCs transfected with the CRISPR-pdCas9-DNMT3a constructs showed an overall increase in methylation of the promoter of *Id2* (*p* = 0.008) and an increased trend in methylation of the promoter of *Id4 (p* = 0.06) compared to cells transfected with the catalytically inactive DNMT3a construct targeted to the same sites (Fig. 3B). Furthermore, reduced expression levels of the target genes were observed in cells transfected with the active DNMT3a vector compared to inactive constructs (Fig. 3C). This pattern was not observed for predicted off-target genes of both guide RNAs (Supplementary Fig. 2).

We further assessed the impact of our epigenetic editing approach on oligodendrocyte differentiation by evaluating the cellular morphology of transfected cells. Immunostaining for MBP on day 9 post transfection showed an increased immunoreactive area in cells transfected with the active CRISPR-pdCas9-DNMT3a construct targeting either *Id2* or *Id4* (Fig. 3D). Quantification of the average process length per cell also revealed longer processes compared to the inactive controls (Fig. 3D). Finally, gene expression analysis at the same time point (9 days post transfection) showed a consistent increase in myelin gene (*Mbp, Mag, Mobp*) expression (Fig. 3E).

### Chronically demyelinated MS lesions show altered methylation and expression profiles for both *ID2* and *ID4*

**Fig. 4:**
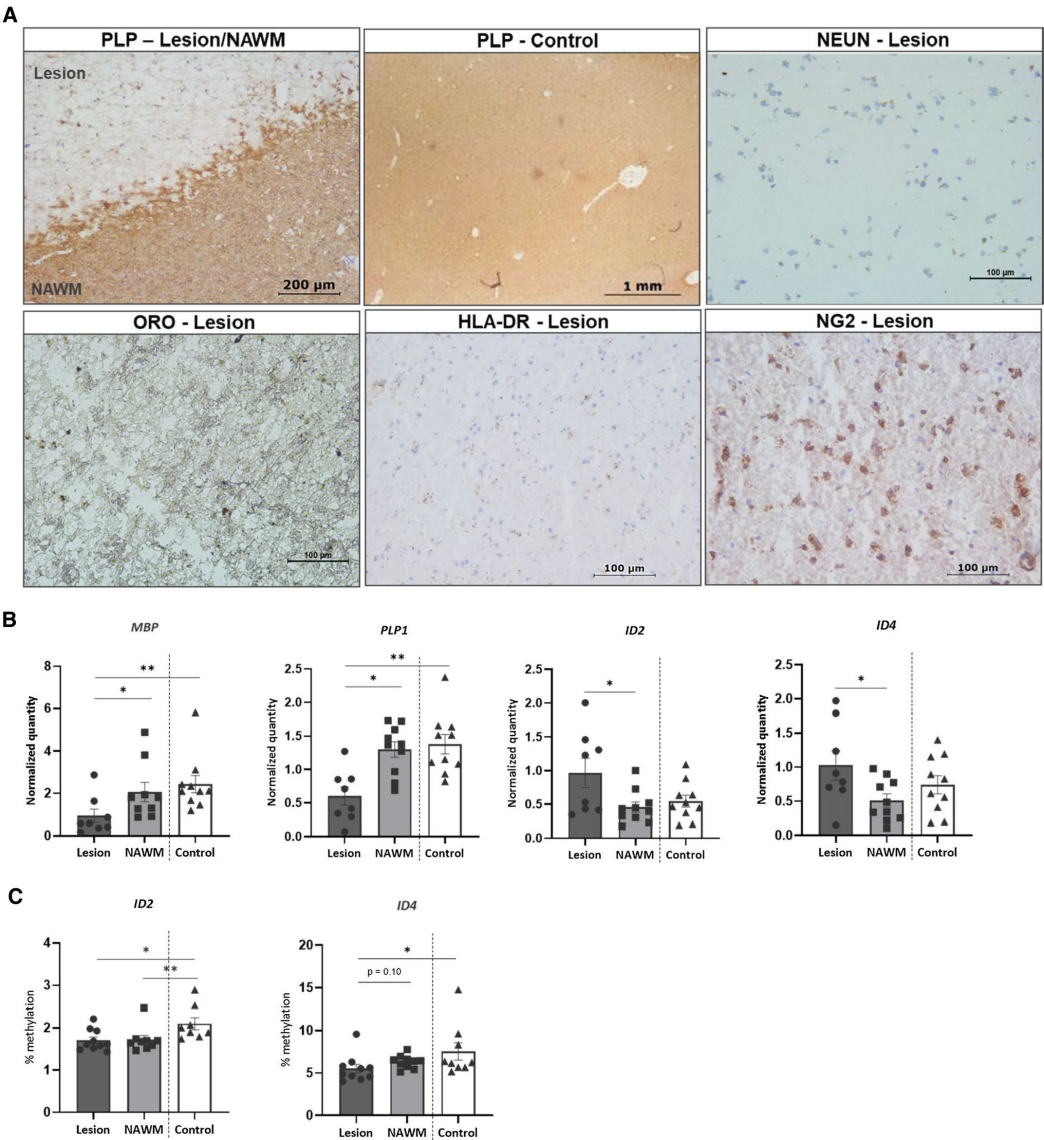
*ID2* and *ID4* are hypomethylated and display increased expression in MS lesions, compared to controls. **A** Phenotyping of MS lesions by means of immunohistochemistry. Chronically demyelinated lesions were defined as PLP^−^, NeuN^−^, ORO^−^, HLA-DR^−^ and NG2^+^. Control white matter samples were defined as PLP^+^, NeuN^−^, ORO^−^, HLA-DR^−^ and NG2^+^. **B** Gene expression analysis of myelin genes (*MBP, PLP*) and the negative regulators (*ID2*, *ID4*) in chronically demyelinated MS lesions, the surrounding NAWM and white matter of matched control samples. Data are corrected for the most stable housekeeping genes (*TBP* and *YWHAZ*), *n* = 8–10, Wilcoxon or Mann–Whitney test. **C** Methylation analysis within the CpG island of the *ID2* and *ID4* promotors in chronically demyelinated MS lesions, the surrounding NAWM and matched control samples (*n* = 10, Wilcoxon or Mann–Whitney test). Data are represented as mean ± SEM, *p < 0.05, **p < 0.01. *NAWM* normal appearing white matter, *PLP* myelin proteolipid protein, *NeuN* hexaribonucleotide binding protein-3, *ORO* Oil red O, *HLA*-*DR* human leukocyte antigen-DR isotype, *NG2* neuron-glial antigen 2, *MBP* myelin basic protein

Since *Id2/Id4* appeared to be epigenetically regulated during murine OPC differentiation, we aimed to examine whether the methylation status of both genes was altered in MS lesions. Progressive MS stages are characterized by the abundance of chronically demyelinated lesions due to impaired endogenous remyelination mechanisms. To assess whether such lesions show differential methylation and/or expression levels of *ID2*/*ID4*, we first phenotyped MS brain lesions to include in our analysis (Fig. 4A). Chronic non-fibrotic demyelinated lesions were characterized based on absence of PLP staining. Further inclusion criteria were the absence of immune cells (HLA-DR^−^, ORO^−^), strictly white matter samples (NeuN^−^), and the presence of OPCs (NG2^+^). Lesions were subsequently microdissected from the surrounding normal appearing white matter (NAWM) and gene expression analysis showed, as expected, reduced level of myelin genes in MS lesions, compared to the surrounding NAWM, and to white matter of age- and sex-matched control samples (Fig. 4B). Interestingly, significantly higher mRNA expression levels of both *ID2* and *ID4* were observed within MS lesions compared to the surrounding NAWM (Fig. 4B). Furthermore, the average DNA methylation levels of both *ID2* and *ID4* were lower in MS lesions compared to control samples (Fig. 4C and Supplementary Fig. 3). Strikingly, particularly ID2 methylation levels within the damage-free NAWM samples followed the pattern observed in the lesions rather than the matched control samples, which suggests that the NAWM might be already affected prior to visible myelin damage (Fig. 4C and Supplementary Fig. 3).

## Discussion

In the present study, we demonstrate that DNA methylation of myelin regulatory genes, in particular the HLH inhibitory transcription factors *Id2* and *Id4*, is crucial for OPC differentiation. Inhibition of DNA methylation in primary OPCs results in significantly increased expression of both genes, eventually inhibiting myelin gene expression and impaired OPC differentiation. Moreover, we show that the decreased expression of *Id2* and *Id4* from OPC to oligodendrocyte stages is negatively correlated with their methylation levels. Our targeted epigenetic editing approach further strengthens evidence for a causal relationship between Id2 and Id4 DNA methylation and OPC differentiation. CRISPR/dCas9-mediated *Id2* and *Id4* hypermethylation resulted in a reduced expression of *Id2* and *Id4*, eventually leading to a boost in OPC differentiation and myelin gene expression. Finally, we show that the promoter regions of *ID2* and *ID4* display decreased methylation in chronically demyelinated MS lesions, resulting in a higher expression of both genes, which could represent a potential key factor in the impaired differentiation capacity of progressive MS patients.

A potential role of DNA methylation enzymes during OPC differentiation and myelination has previously been described [19–22]. Yet, it was unclear which genes are actually epigenetically regulated during oligodendrocyte development. OPC differentiation is featured by the induced expression of myelin genes, which are tightly regulated by an upstream network of activators and repressors [3, 6]. Here, we show that pharmacological inhibition of DNA methylation, by means of 5-AZA, mainly affects the repressive transcription factors *Id2* and *Id4* and prevents OPC differentiation. Even though the use of epigenetic drugs has its limitations, such as the lack of specificity and relatively high cytotoxicity, it can provide more insights into DNA methylation biology [25, 26]. While we cannot exclude the possibility that other relevant genes may have been affected by the use of 5-AZA, there is evidence from previous studies that show similar effects on OPC differentiation and *ID4* expression when using epigenetic modifiers, such as HDAC inhibitors [16, 27]. *Id2* and *Id4* belong to the HLH transcription factors and are highly expressed in OPC stages, a notion confirmed in our current study. Both inhibitory proteins function to keep OPCs in a non-differentiated stage by antagonizing the nuclear translocation of pro-differentiation HLH transcription factors (OLIG1/2, ASCL1) [28, 29]. Indeed, we show that the expression of *Id2*/*Id4* significantly decreased in differentiated oligodendroglial stages. Interestingly, the expression profile of *Id2*/*Id4* was negatively correlated with the methylation profile of the respective promoter regions, thereby suggesting that DNA methylation could represent the mechanism of action behind this stage-dependent regulation. This is in line with previous observations that show that the type II protein arginine methyltranferase PRMT5 associates with the CpG islands of *Id2* and *Id4* and thereby regulating their expression during OPC differentiation [30].

Even though both *Id2* and *Id4* seem to be epigenetically regulated during OPC differentiation, there was still no functional evidence that specific alterations to the methylation profile of the genes will influence oligodendroglial development. To assess this intricate causality, we made use of a recently developed epigenetic engineering system, based on CRISPR-Cas9 technology. Recent advantages have been made to alter the epigenome in a targeted manner, by coupling the nuclease-inactivated dCas9 protein to epigenetic editor domains (such as DNMT3a and TET1). Target-specificity is then achieved by designing a guide RNA towards the desired CpG region [31–33]. For this study, we used the plasmid vector designed by Vojta et al. [24]. The dCas9 protein was coupled to the catalytic domain of DNMT3a, which allows for targeted methylation of *Id2* or *Id4*, based on the guide RNA that was cloned into the vector. As a control, we took along the DNMT3a-inactive plasmid, which had the same properties as the active vector, but lacked the capacity to induce methylation. Transfection of primary OPCs with the *Id2* or *Id4* plasmids led to higher methylation levels at the targeted region, accompanied by a reduced expression of both genes at day 6 post transfection. This timing was specifically chosen, as it has been shown that the peak of methylation is expected to be at the highest point between day 6 and 7 post transfection [24]. However, it must be noted that the methylation levels within the *Id4* promoter region are difficult to measure due to the dense CpG-rich regions, which limits the options for adequate primer design. We only measured the methylation level of seven CpG sites of the *Id4* promotor region due to our limited possibilities in primer design. It might still be possible that other relevant CpG sites show higher changes in % methylation following CRISPR-Cas9. In general, the overall increase in methylation follows our line of expectancy and does nicely show that our CRISPR-Cas9 vector does induce methylation at the targeted regions. Most interestingly, we also observed a similar increase in the expression of myelin genes and boost in OPC differentiation (MBP area and process length) at day 9 post transfection when targeting either *Id2* or *Id4*, thereby validating the functional importance of DNA methylation of *Id2/Id4* during oligodendrocyte development. However, since we observed a significant increase in *Mag* in both *Id2* and *Id4* targeted samples, and a non-significant increase in *Mbp* and *Mobp*, we assume that the cells were still differentiating at the time of lysation and did not reach the ultimate final stage yet. Interestingly, even though both *Id2* and *Id4* have a similar effector function in the regulation of OPC differentiation, targeted silencing of only one of the genes was already sufficient to boost the expression of myelin genes. This is in line with previous literature, that has shown that both *Id2* and *Id4* function separately as an intracellular timer for oligodendrocyte differentiation. Absence of *Id2* results in premature OPC differentiation and a higher percentage of oligodendrocytes. Similarly, overexpression of *Id4* in OPCs increases their proliferation and inhibits their differentiation into oligodendrocytes [34].

CRISPR-Cas9-based epigenetic editing has gained increasing attention because of its ease-of-use and rapid adaptability. However, one main concern remains the high off-target effects due to the complementarity of the guide RNA with other genomic regions. Even though we did not observe significant predicted off-target effects, we cannot completely rule out misguided dCas9-DNMT3a events. A study has previously shown that the dCas9-DNMT3a tool increases the methylation levels globally, regardless of the use of guide RNAs [35]. It is thus suggested that the unspecific activity of epigenetic editing tools is not only a result of off-target guide RNA binding, but also unguided activity of the effector domains, such as DNMT3a, themselves [36, 37].

Since we have shown that *Id2* and *Id4* are epigenetically regulated during normal OPC differentiation, we wondered whether these processes were affected in pathological conditions. MS represents one of the major myelopathies of the CNS and is characterized by early endogenous remyelination, a process that becomes impaired during the progressive stages of the disease [8, 38]. It has been suggested that the main reason behind this hampered remyelination is a block in OPC differentiation within MS lesions [39]. We therefore hypothesized that the methylation profile of *ID2* and *ID4* was altered in chronically demyelinated MS lesions, and thus could represent one of the reasons behind the differentiation block. MS lesions are typically very diverse in terms of the degree of demyelination, inflammation and scar formation [40, 41]. In the present study, we aimed to include only chronically demyelinated lesions which are inflammatory inactive. These lesions are mostly found in progressive MS patients and represent the main neurodegenerative aspect of the disease. Other important criteria that we applied in our study were the presence of OPCs within the lesions and the exclusion of scar tissue since these have no ability to regenerate and are too advanced in the disease stage. Gene expression analysis showed higher expression of both *ID2* and *ID4* within the lesions, compared to the surrounding NAWM. Even though this observation is in line with our hypothesis this difference could represent the balance between the presence of OPCs within the lesions and oligodendrocytes within the NAWM. However, we also showed that the promoter regions of both *ID2* and *ID4* were hypomethylated within MS lesions, compared to age- and sex-matched controls. Furthermore, the average methylation pattern of ID2 within the NAWM samples resembled the methylation pattern of the lesions, rather than the non-neurological control samples. This suggests that there could already be some OPC dysregulation occuring within MS brains preceding noticeable myelin damage, a notion that has been proposed before by others [42, 43]. Interestingly, the average expression levels of both genes was substantially lower in the control cohort compared to the lesions, yet not statistically significant. This could be attributed to multiple aspects, such as the variation between healthy individuals, the RNA integrity of the samples due to the variation in post-mortem interval, or the lack of statistical power due to the low sample size.

Our data demonstrate that chronically demyelinated lesions show dysregulation of *ID2* and *ID4* both on the level of methylation and gene expression, which could be an underlying mechanisms behind the OPC differentiation block in progressive MS stages. Our observations are in line with previous research, which has shown that chronic MS lesions show higher histone acetylation levels, associated with an increase in the expression of ID2 and TCF7L2 [44]. OPCs have recently also been described as environmental biosensors that can alter their epigenomic signature in response to chemical and physical stimuli, such as neuronal activity, stiffness of the extracellular matrix and the prescence of hormones [45]. In line with this rationale, our data could suggest that the accumulation of myelin damage during disease progression induces a change in the epigenetic regulation of *ID2/ID4*, thereby leaving the cells in a blocked differentiation stage. A limitation is that our findings are based on heterogenous bulk tissue, and therefore the presence of other cell types may bias the observed changes in methylation. For example, it has previously shown that Id4 is necessary for astrocyte proliferation after excitotoxic damage, while Id2 has been shown to be upregulated in specific microglia clusters, associated with aging [46, 47]. The cell-type heterogeneity within bulk tissue can thus confound analysis and lead to data misinterpretation. Over the recent years, new in silico methods have been developed to estimate cell-type proportions within bulk tissue for the analysis of epigenome data [48, 49]. However, such cell-type deconvolution algoritms are not yet applicable for targeted DNA methylation analysis. Especially complex tissues, such as MS brain lesions, of which the cellular composition is very variable and hard to correct for, should therefore be considered with care. Furthermore, the methylation profile does not only differ between different cell types, but can also vary strongly within one cell population, mainly in a pathological context. Indeed, recent studies have revealed distinct OPC and oligodendrocyte populations within MS brain samples, each with different transcriptional, and likely epigenetic, signatures, which could therefore result in misinterpretation of bulk tissue analysis (50, 51). Nevertheless, our observations regarding *ID2/ID4* methylation within MS brain lesions are in line with our previous in vitro findings.

Taken together, our study reveals the epigenetic regulation of the inhibitory transcription factors ID2 and ID4 during OPC differentiation. Furthermore, this epigenetic signature appears to be dysregulated in chronically demyelinated MS lesions. Our data provide more insights into OPC biology, while also unraveling new epigenetic targets to boost OPC differentiation that appears relevant in the context of MS.

## Supplementary Information

Below is the link to the electronic supplementary material.

Supplementary file1 (DOCX 380 KB)

## Data Availability

All relevant data are within the manuscript and Supplementary Information Appendix.
