# Supplementary material for: DNA methylation regulates the expression of the negative transcriptional regulators ID2 and ID4 during OPC differentiation
Source: Cell Mol Life Sci. 2021 Sep 5;78(19-20):6631–44. doi: 10.1007/s00018-021-03927-2 (PMC8558293; doi:10.1007/s00018-021-03927-2)
Supplement: Supplementary file 1 — Supplementary file1 (DOCX 380 KB) [file 18_2021_3927_MOESM1_ESM.docx]

**Supplementary Tables**

**Table 1:** **Guide RNA targeting *Id2* and *Id4***


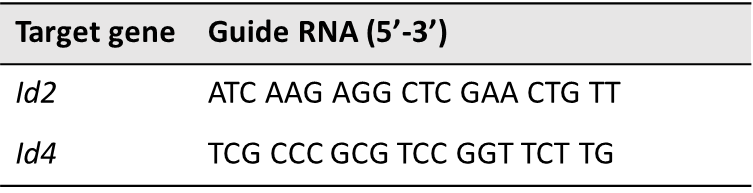


**Table 2: Antibody list**


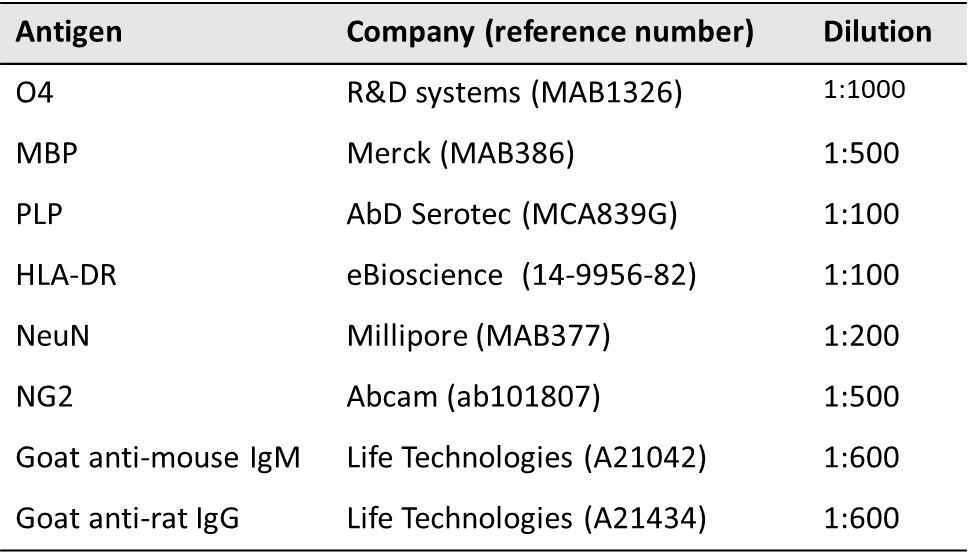


**Table 3: qPCR primer list**


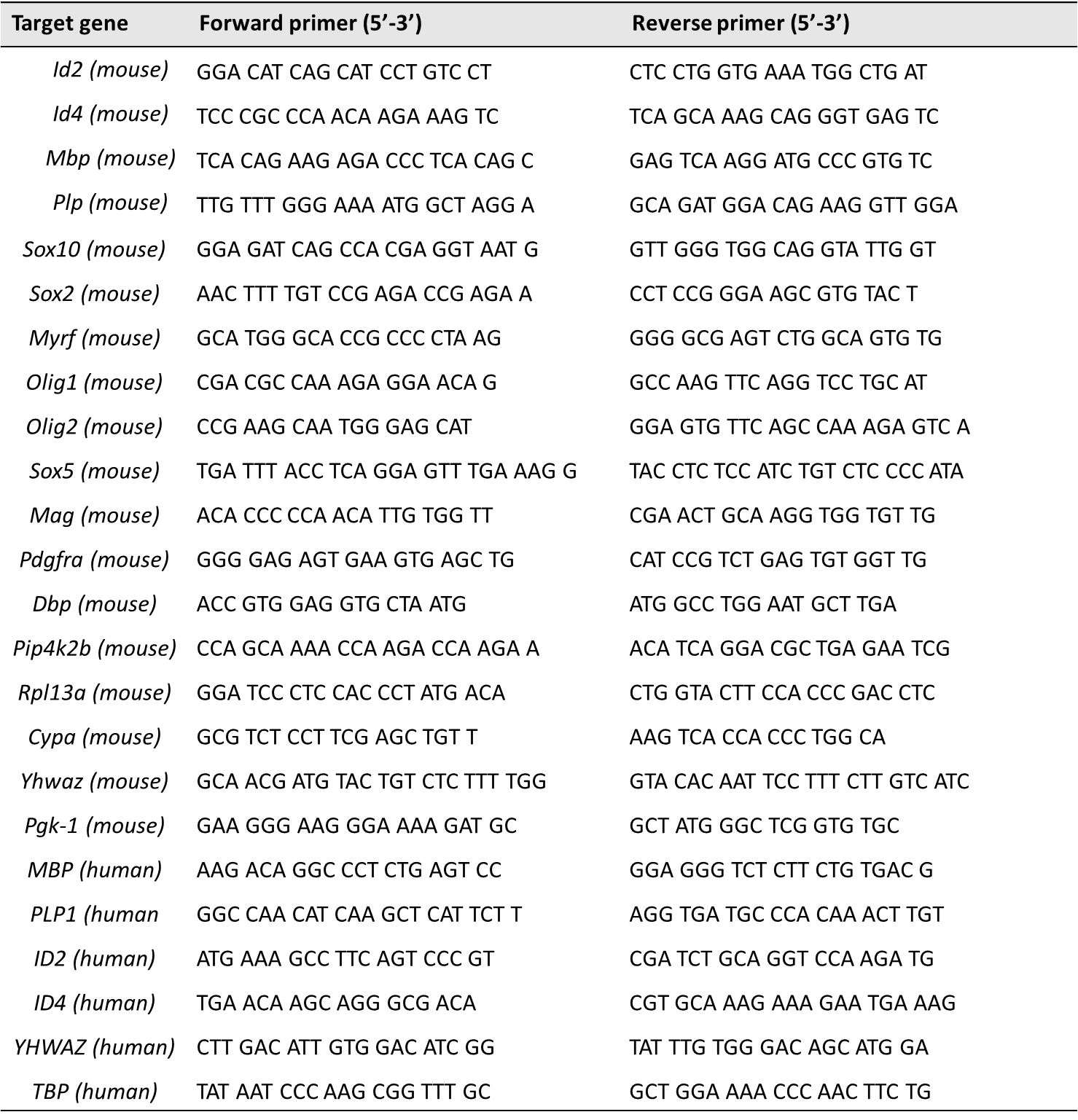


**Table 4: Pyrosequencing primer list**


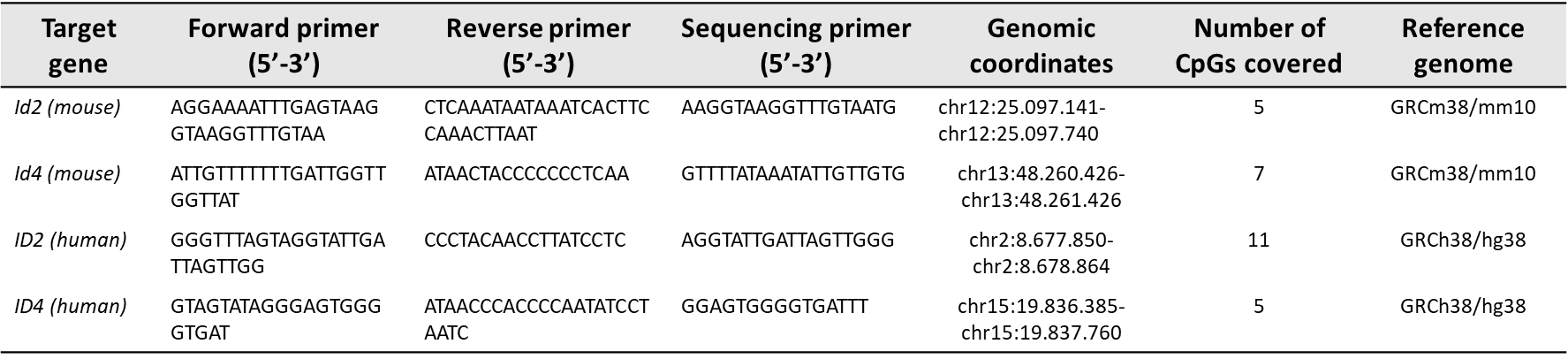


**Supplementary Figures**

**Figure S1 – Treatment with 5-AZA does not affect Oli-*neu* cell viability at a concentration of 1 µM.** Oli-*neu* cells were treated with different concentrations of 5-AZA for 48 hours. DMSO was used as a vehicle control condition. Cell viability was assessed via an MTT-assay. Data are represented as mean + SEM and are relative to the control condition (n = 6; **p<0.01, ***p<0.001, ****p<0.0001).

**
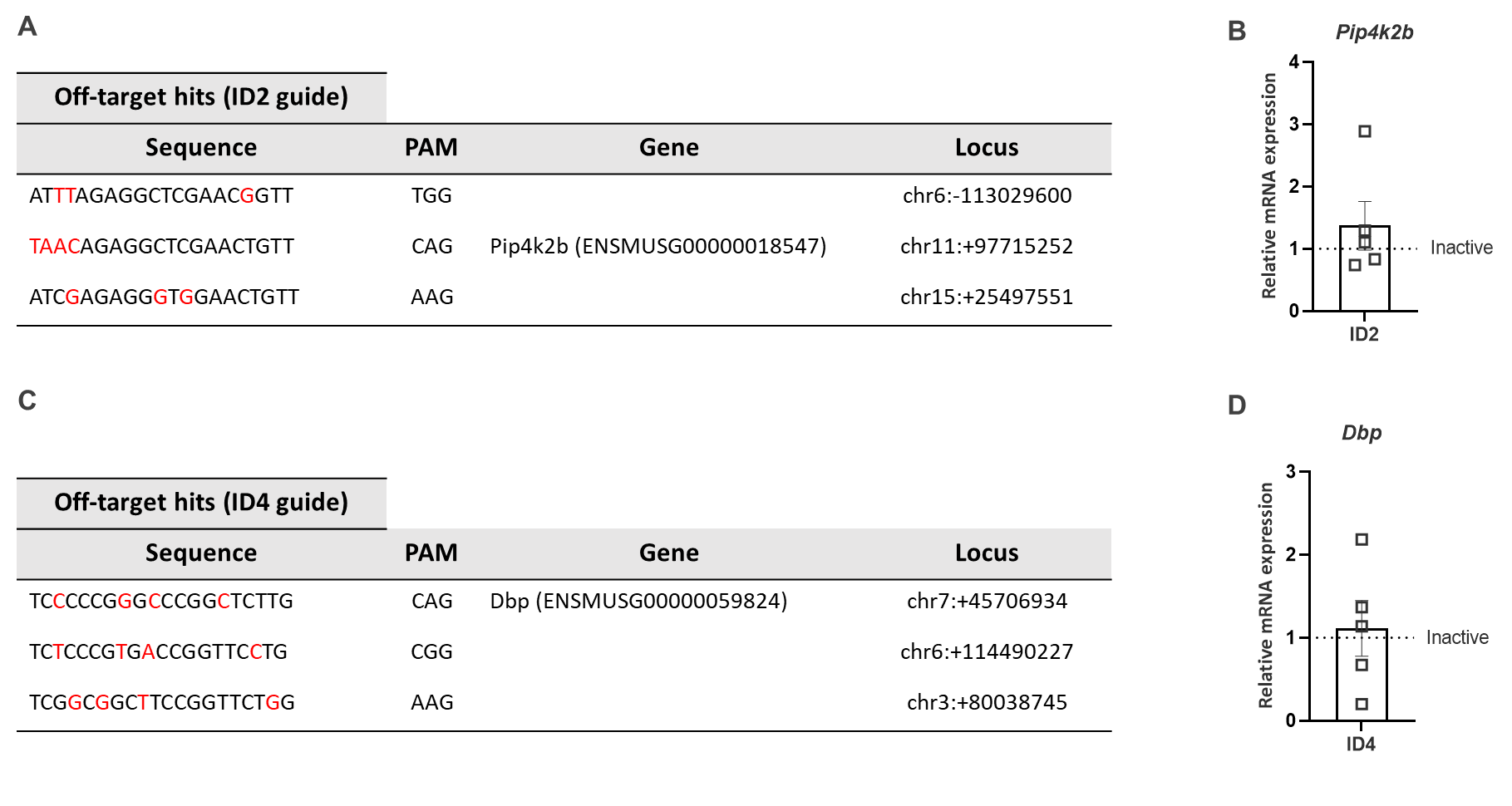
**

**Figure S2 – Top 3 off-target hits of the *Id2/Id4* sgRNA.** (A,C) The most likely off-targets of the designed sgRNAs are determined by the Benchling software®. The mismatches are depicted in red. (B,D) Off-target effects are analyzed by qPCR of the relevant genes. No difference between the active and inactive constructs is observed. Data are represented as mean ± SEM, n=5 (one sample t-test).

**
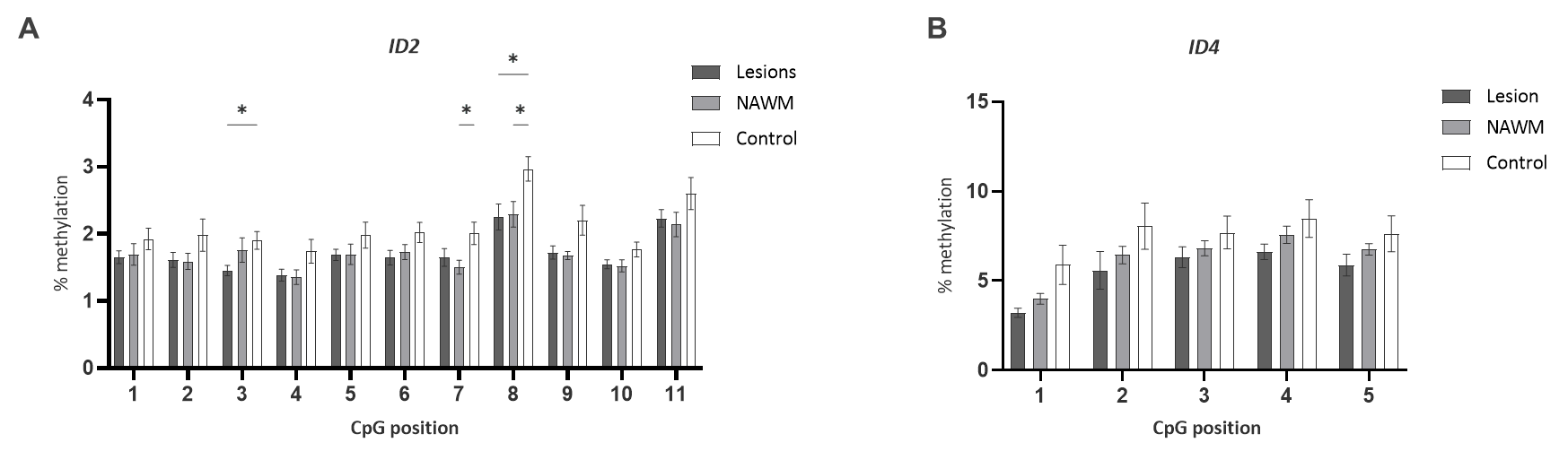
**

**Figure S3 – *ID2* and *ID4* methylation levels per measured CpG site.** Methylation analysis within the CpG island of the *ID2* and *ID4* genes in chronically demyelinated MS lesions, the surrounding NAWM and matched control samples (n=10, two-way repeated measures ANOVA with Šídák's multiple comparisons test). Data are represented as mean ± SEM, *p<0.05.

**Supplementary Methods**

## Cell viability assay

The effect of 5-AZA on cell viability and cell survival was measured via the 3-(4, 5-dimethylthiazolyl-2)-2,5-diphenyltetrazolium bromide (MTT) assay. Oli-*neu* cells were seeded in a 96-well plate at a density of 20 x 10^3^ cells per well in standard culture medium (DMEM, 10% FCS, 1% P/S). Cells were treated with increasing concentrations of 5-AZA or dimethylsufloxide (DMSO; Sigma-Aldrich) as a control. After 48 hours, medium was removed and cells were incubated with 500 µg/ml MTT (Sigma-Aldrich) in DMEM for four hours at 37°C. After removal of the MTT-solution, a glycine-DMSO mixture was added to induce reduction of MTT to formazan. The absorbance was measured at 540 nm with the iMark Microplate Reader (Bio-rad Laboratories, Temse, Belgium).
